# The Use of a Simple Vaginal Speculum to Harvest Quadriceps Tendon Autografts for Anterior Cruciate Ligament Reconstruction and Avoid Any Potential Pitfalls during Harvesting Procedure: Technical note

**DOI:** 10.18295/squmj.1.2024.004

**Published:** 2024-05-27

**Authors:** Nikolaos E. Koukoulias, Angelo V. Vasiliadis, Theofilos Dimitriadis

**Affiliations:** Department of Orthopaedic Surgery - Sports Trauma Unit, St. Luke’s Hospital, Thessaloniki, Greece

**Keywords:** Anterior Cruciate Ligament, Knee, Arthroscopic Surgery, Quadriceps Muscle, Autografting, Greece

## Abstract

This technical note aimed to present a straigthforward method for harvesting quadriceps tendon autografts with the use of a simple vaginal speculum and direct visualisation of a scope. Anterior cruciate ligament reconstruction with quadriceps tendon autografts has gained popularity in recent years, with many harvesting techniques that use different harvesting systems available on the market. These techniques vary from transverse to longitudinal skin incisions and from open to minimally invasive approaches and have a learning curve, as with the majority of surgical procedures. The technique proposed in this technical note is minimally invasive, can be easily reproduced by any surgeon irrespective of their experience, has a short learning curve, requires no additional cost or technical support during the procedure and creates a stable working space that allows for freedom of manipulation of surgical instruments and the arthroscope.

Quadriceps tendon (QT) is a promising alternative autograft used for anterior cruciate ligament reconstruction (ACLr).^1^ Different techniques can be employed for harvesting QT, using either an open or minimally invasive approach and full or partial thickness grafts, with or without bone block and/or QT defect repair.^1^^,^^2^ The Danish Knee Ligament Reconstruction Registry—a prospective, nationwide web-based study—analysed the data of 16,579 ACLr and demonstrated a higher revision rate of QT grafts, compared to hamstring tendon (HT) and patellar tendon grafts. The different levels of experience among the multiple surgeons involved in these techniques and the higher revision rates confirmed by low-volume clinics that have fewer than 100 procedures per year might have been associated with those results.^3^^,^^4^ Additionally, previous studies have reported that QT graft harvest is a technically demanding procedure that involves a steep learning curve when harvesting the autograft, requiring the use of different harvesting tools available on the market.^5^^,^^6^

## Surgical Indications and Contraindications

The indications for ACLr with the use of QT grafts appear to be similar to those for other autografts (HT and bone-patellar tendon-bone [BPTB] grafts) and include young active patients (especially athletic patients aged <25 years), complex knee ligament injuries and revisions cases. However, possible contraindications include, but are not limited to, patients with chronic quadriceps tendinopathy, quadriceps muscle atrophy, a history of prior tendon rupture and patella fractures.^7^^,^^8^

## Advantages and Disadvantages of Quadriceps Tendon Grafts

QT autografts offer many advantages over HT and BPTB grafts. First, QT grafts are associated with a lower rate of anterior knee pain/pain with kneeling and less sensation of numbness, when compared to BPTB and HT grafts, respectively.^5^^,^^7^ Second, histologically, QT grafts can provide approximately 20% more collagen fibres, higher fibril-interstitium ratio and higher fibroblast density per cross-sectional area, when compared to BPTB grafts.^6^ Third, the graft thickness in QT grafts is more predictable than that in HT grafts.^6^ QT grafts also provide the advantage of preserving the hamstring strength and dynamic stability of the knee [Table 1]. This has been linked to reduced ACL injuries, as these grafts prove crucial when treating professional soccer players who have suffered ACL tears.^9^

Most disadvantages of QT grafts are associated with loss of orientation during their harvesting, which may challenge an inexperienced surgeon in identifying the medial and lateral borders of the QT.^10^ Potential drawbacks of this approach may also be associated with harvesting of inappropriate graft length and lateralised or medialised splitting of the donor QT. Skin necrosis due to excessive tension of the wound from the vaginal speculum is also possible, especially in the case of small skin incisions. This is associated with the placement of the vaginal speculum with the knee in extension, followed by the placement of the knee in 90 degrees of flexion, thus raising the tension of the skin. Finally, the haematoma formation at the musculotendinous junction of the quadriceps (more common with the use of full-thickness graft) and the risk of patella fracture (with the use of bone block technique) are also possible drawbacks of this harvesting method.^6^^,^^10^

## Current Harvesting Techniques

Several different techniques used for the ACLr employ QT as an autograft, including open or minimally invasive methods for harvesting and the use of full or partial-thickness grafts, with or without patellar bone blocks.^1^^,^^2^ Fink *et al*. performed a transverse skin incision over the superior border of the patella and exposed the QT subcutaneously by using a long Langenbeck.^11^ In contrast, Malinowski *et al*. performed a 4- to 5-cm skin incision in a sagittal plane perpendicularly to the superior pole of the patella, starting 4 to 5 cm proximally to the patella and finishing distally, specifically 1 cm over the patellar dorsal surface. In their harvesting technique, the authors used two Farabeuf retractors to visualise the QT and its medial and lateral border.^10^ Alternatively, Ollivier *et al*. moved the knee from a flexed to an extended position and exploited skin elasticity (the technique of movable window) to harvest the QT bone graft by direct vision. To improve visualisation, they used two retractors at the proximal part of the incision to apply strong traction on the bone graft distally.^5^

## Novelty of the New Technique

The present technical note attempts to address the challenges associated with QT harvesting (loss of orientation during the harvesting procedure) while minimising the risk of possible graft failure and revision rates in patients who undergo ACLr with QT grafts. The dry and direct endoscopic view of the QT, with the use of a simple vaginal speculum, facilitates the visualisation of the *vastus medialis* and *vastus lateralis* muscle bellies of the QT, as well as the initial cutting point from proximal to distal. To the best of the authors’ knowledge, this is the first report that describes the use of a simple vaginal speculum to assist in the harvesting of QT grafts and limit possible complications during the harvest.

## Surgical Technique

A 42-year-old male patient presented to the orthopaedic department of a tertiary care hospital in Thessaloniki, Greece in 2022. The patient was placed in a supine position, with two posts attached to the operation table to facilitate access for the surgeon. The first post was lateral to the proximal thigh, while the second was used as a footrest to maintain the knee position in 90 degrees of flexion. After anaesthesia induction, the operative leg was prepared and draped in a sterile fashion. Then a tourniquet was placed in a standardised location on the thigh and inflated to 250 to 300 mm Hg.

**Figure 1:**
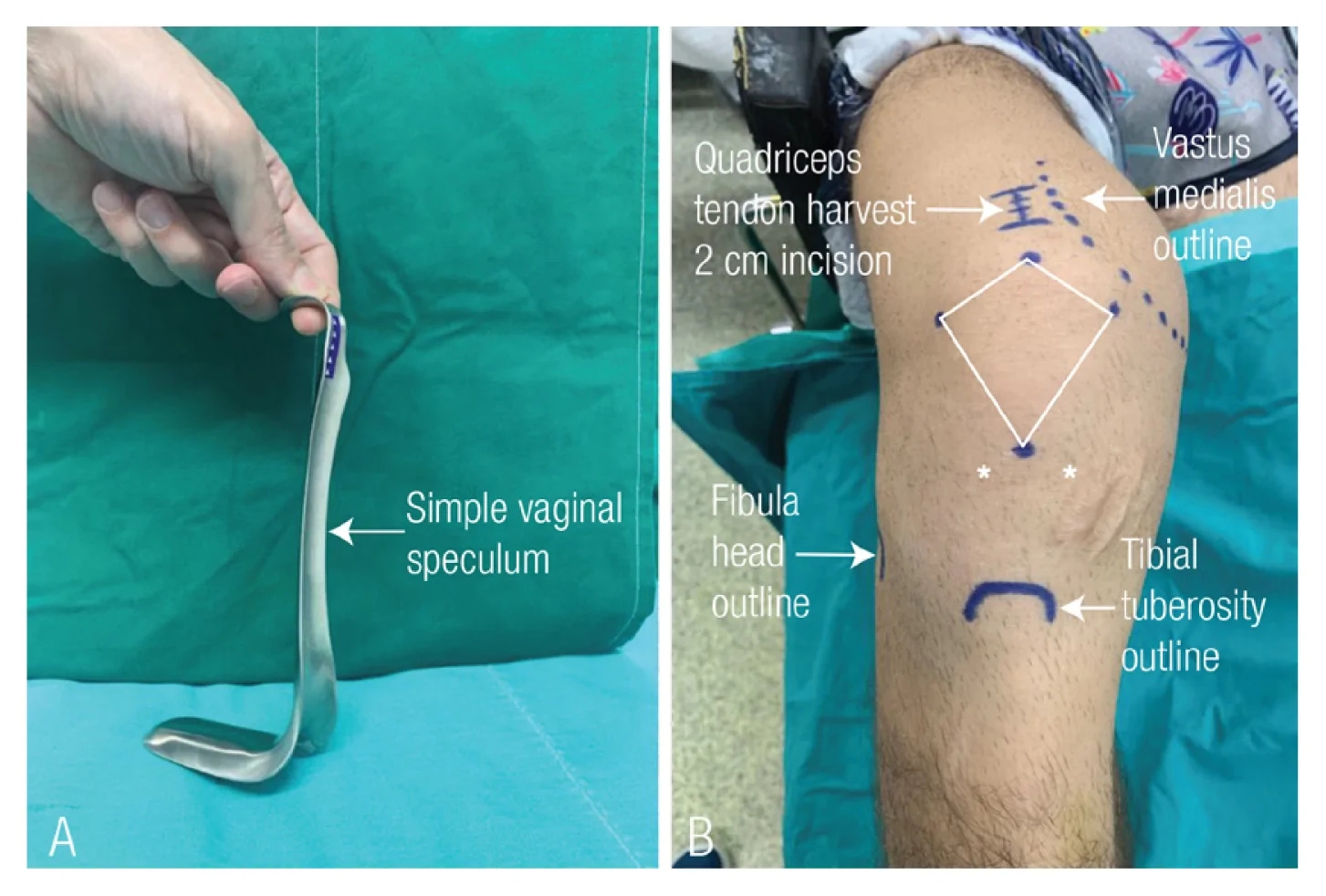
Intraoperative photographs showing **(A)** a simple vaginal speculum used to retract the anterior skin and soft tissue and **(B)** the planned surgical skin incision for quadriceps tendon harvest. A 2-cm longitudinal skin incision is needed to allow access to the quadriceps tendon, which extends proximal from the superior pole of the patella (quadrilateral). The standard arthroscopic portals (asterisk) were used during the anterior cruciate ligament reconstruction.

**Figure 2:**
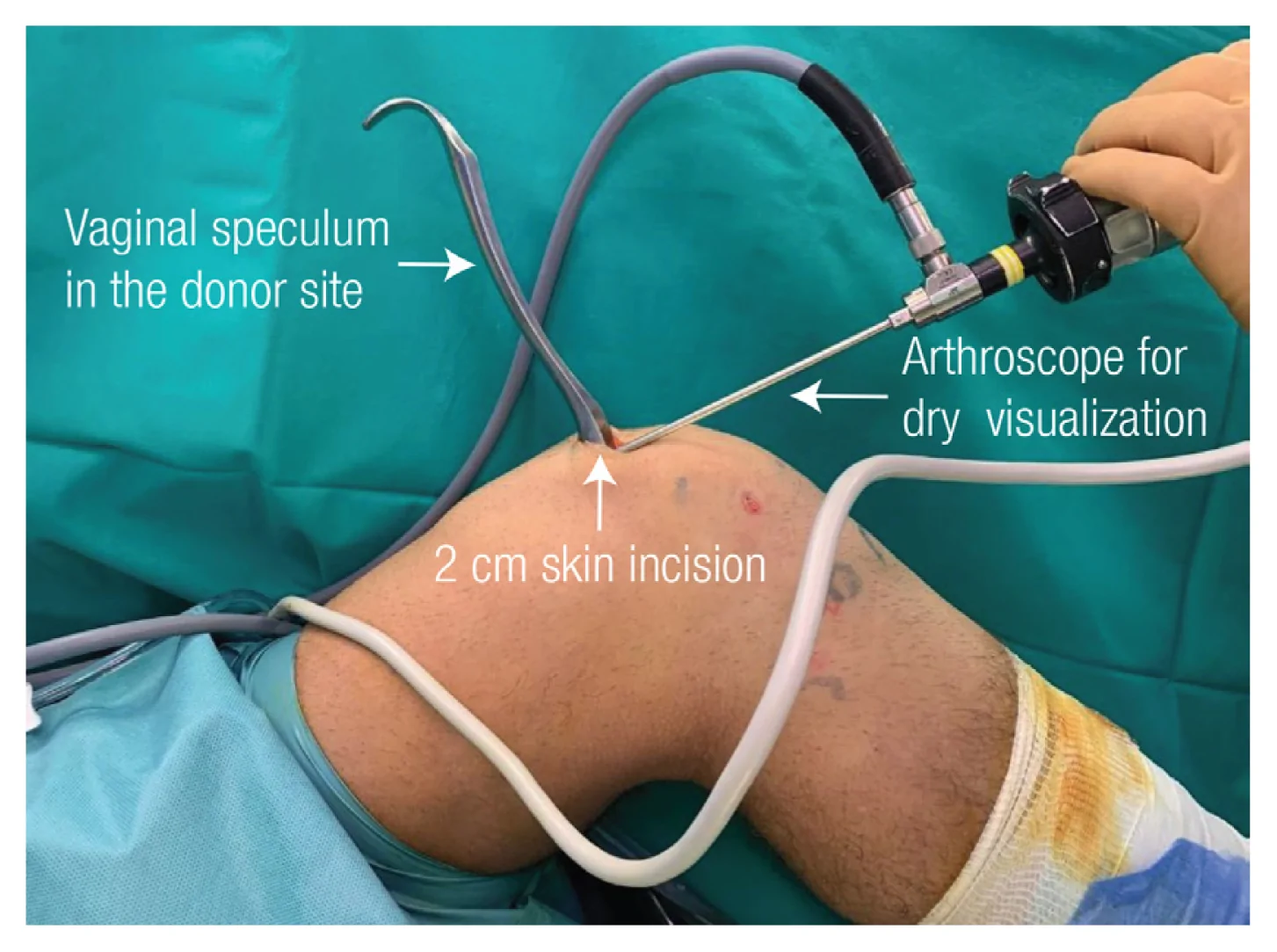
Intraoperative photograph of the right knee showing the donor site with a dry arthroscopic camera.

**Figure 3:**
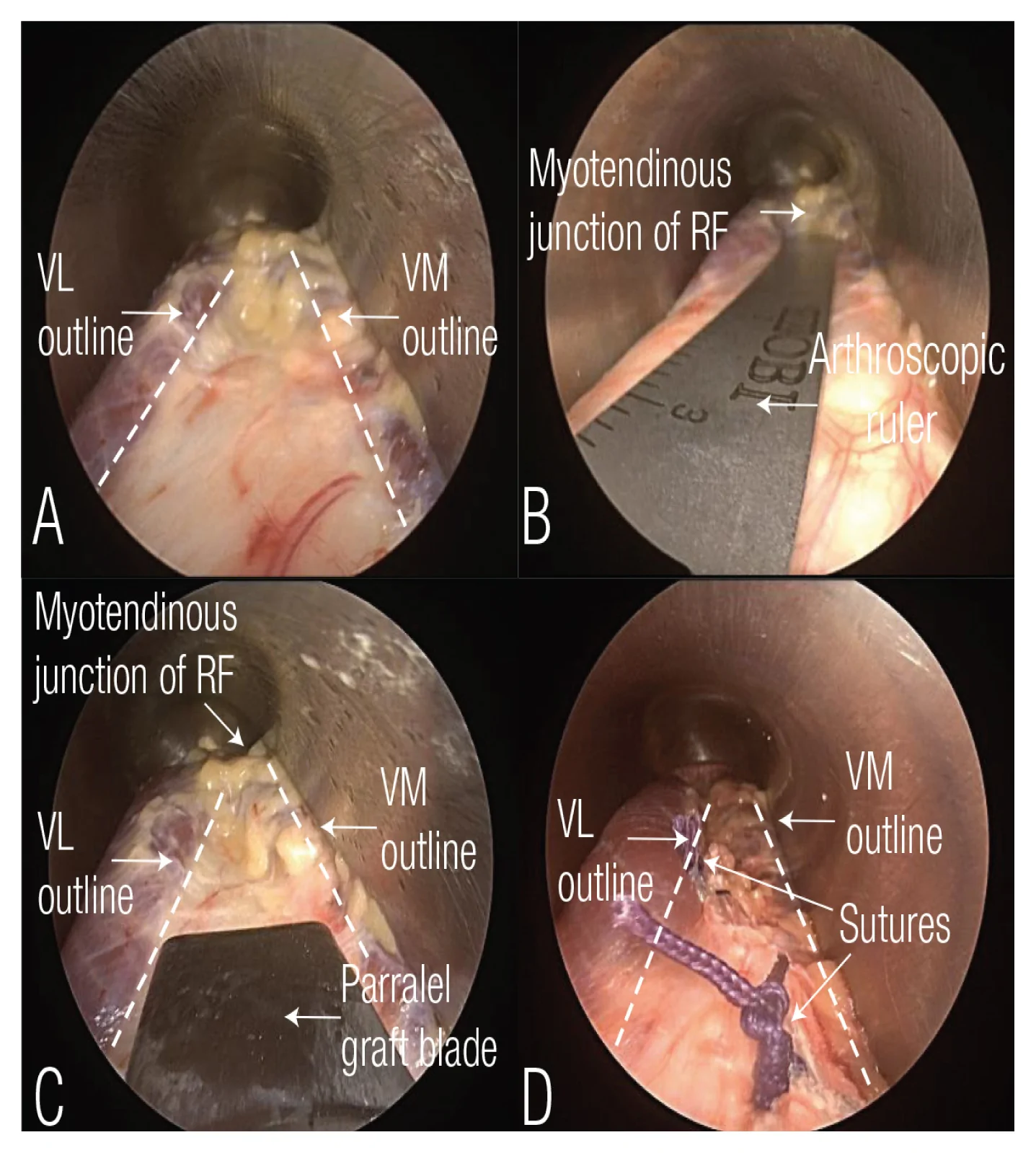
Arthroscopic images of direct endoscopic visualisation of quadriceps tendon showing **(A)** the whole length and borders of quadriceps tendon, **(B)** the measuring and **(C)** harvesting of the graft with a parallel graft blade (Arthrex 10 mm tendon stripper blade). The full-thickness tendon defect is repaired with No. 2 non-absorbable sutures with the use of the Scorpion Suture Passer (Arthrex, Naples, Florida, USA); **(D)** the gap closure was confirmed endoscopically, and the scope is introduced above the harvesting site of the graft. VL = vastus lateralis; VM = vastus medialis; RF = rectus femoris.

The use of a vaginal speculum was used to retract the anterior skin and thigh soft tissue, visualise the borders, measure the length of the QT, harvest the graft and perform side-to-side defect closure [Figure 1]. A 2-cm longitudinal skin incision was made 1 cm proximal to the superior pole of the patella and carried down through the subcutaneous tissue and deep fascia until the QT is visualised. The soft tissues were easily released using a finger for better visualisation of the tendon. Then, the simple vaginal speculum was inserted and used as a retractor of the anterior skin and subcutaneous tissue to enable dry arthroscopy and fully expose the borders of the QT [Figure 2]. At this point, the use of a gauze pad can facilitate the removal of any remaining soft tissue, allowing for a better view of the tendon. Once minimum space was developed over the QT, dry arthroscopy was utilised to visualise the whole length and borders of QT, harvest tendon graft and perform side-to-side defect closure [Figure 3]. It is important to note that the graft was harvested by applying firm tension on the distal sutures, while the QT stripper cutter was used to strip and cut the autograft proximally once the desired graft length was achieved (Arthrex, Naples, Florida, USA) [Figure 4]. The simple vaginal speculum served as a tent, while the surgeon identified the proximal myotendinous junction of the *rectus femoris* and the starting point of the graft harvested in the proximal part of the patella, where the skin incision was made. Ultimately, the entire length of the QT was visualised. Based on the available length of the tendon, the length and width of the graft to be harvested depends on the desired size of the tendon graft. Additionally, the skin incision serves both as a viewing and working portal [Supplementary Video 1]. Consent for publication purposes was obtained from the patient.

## Conclusion

A major advantage of this technique is the provision of a direct view of the entire border of the QT for the surgeon during harvesting and, therefore, the minimalisation of technical errors. This technique can be easily performed by all surgeons, regardless of their level of experience as it does not have a steep learning curve. The required instrumentation consists of a simple vaginal speculum, a reusable instrument, without additional cost for the procedure. This instrument does not rely on the availability of the product and does not require any technical support. This technique can also easily reproduce the harvesting method of QT graft for the ACLr by creating a stable working space and providing freedom of manipulation of the surgical instruments and arthroscope.

## Supplementary Information



## Figures and Tables

**Table 1:** Advantages and disadvantages of the reported harvesting technique

Advantages
Minimally invasive with a 2-cm longitudinal skin incision
No need for extra assistance
Reproducible irrespective of the surgeon’s level of experience
Short learning curve
Creates a stable working space that provides freedom of manipulation of the surgical instruments and arthroscope
Lower rate of anterior knee pain
Predictable graft thickness
Preservation of hamstring strength
**Disadvantages**
Potentially more time-consuming
Care must be taken to visualise the tendon borders
Skin necrosis may occur due to excessive tension of the wound (for small skin incisions)
Patella fracture (with bone block technique)
A tourniquet has to be used
